# A Transgenic Model Reveals the Role of Klotho in Pancreatic Cancer Development and Paves the Way for New Klotho-Based Therapy

**DOI:** 10.3390/cancers13246297

**Published:** 2021-12-15

**Authors:** Tammi Arbel Rubinstein, Inbal Reuveni, Arkadi Hesin, Anat Klein-Goldberg, Hannes Olauson, Tobias E. Larsson, Carmela R. Abraham, Ella Zeldich, Assumpció Bosch, Miguel Chillón, Kenneth Samuel Hollander, Ayelet Shabtay-Orbach, Gilad W. Vainer, Ido Wolf, Tami Rubinek

**Affiliations:** 1 Institute of Oncology, Tel Aviv Sourasky Medical Center, Tel Aviv 64239, Israel; rtammi@gmail.com (T.A.R.); inbalreu1@gmail.com (I.R.); arkadih@tlvmc.gov.il (A.H.); anatkgoldberg@gmail.com (A.K.-G.); kennethh@tauex.tau.ac.il (K.S.H.); ayelet_shabtay@yahoo.com (A.S.-O.); idow@tlvmc.gov.il (I.W.); 2 Sackler Faculty of Medicine, Tel Aviv University, Tel Aviv 6997801, Israel; 3 Division of Renal Medicine, Department of Clinical Science, Intervention and Technology, Karolinska Institutet, 14186 Stockholm, Sweden; hannes.olauson@gmail.com (H.O.); tobias.larsson@ki.se (T.E.L.); 4 Department of Nephrology, Karolinska University Hospital, 17176 Stockholm, Sweden; 5 Boston University School of Medicine, Department of Biochemistry, Boston, MA 02118, USA; cabraham@bu.edu (C.R.A.); ezeldich@bu.edu (E.Z.); 6 Pharmacology & Experimental Therapeutics, Boston, MA 02118, USA; 7 Klogenix Therapeutics Inc., Boston, MA 02116, USA; Assumpcio.Bosch@uab.cat (A.B.); Miguel.Chillon@uab.cat (M.C.); 8 Institut de Neurociències, Department of Biochemistry and Molecular Biology, Universitat Autonoma de Barcelona, 08035 Barcelona, Spain; 9 Unitat Mixta UAB-VHIR, Vall d’Hebron Institut de Recerca (VHIR), 08035 Barcelona, Spain; 10 CIBERNED, Instituto de Salud Carlos III, 28029 Madrid, Spain; 11 ICREA, Institut Catalan Recerca Avançada, 08010 Barcelona, Spain; 12 Pathology Institute, Tel Aviv Sourasky Medical Center, Tel Aviv 6423906, Israel; giladwv@gmail.com

**Keywords:** klotho, KL1, sKL, tumor suppressor, pancreatic cancer, PDAC

## Abstract

**Simple Summary:**

We aimed to study the role of the anti-aging protein klotho and its secreted isoform, sKL, in pancreatic cancer. Three in vivo models, including a novel genetic mouse model and bioinformatics analyses, indicated klotho as a tumor suppressor in pancreatic ductal adenocarcinoma, and unveiled a unique klotho DNA hypermethylation pattern in pancreatic tumors. These results possess significant prognostic value, and further suggest that sKL may serve as a therapeutic agent for pancreatic ductal adenocarcinoma.

**Abstract:**

Klotho is an anti-aging transmembrane protein, which can be shed and can function as a hormone. Accumulating data indicate that klotho is a tumor suppressor in a wide array of malignancies, and designate the subdomain KL1 as the active region of the protein towards this activity. We aimed to study the role of klotho as a tumor suppressor in pancreatic ductal adenocarcinoma (PDAC). Bioinformatics analyses of The Cancer Genome Atlas (TCGA) datasets revealed a correlation between the survival of PDAC patients, levels of klotho expression, and DNA methylation, and demonstrated a unique hypermethylation pattern of klotho in pancreatic tumors. The in vivo effects of klotho and KL1 were examined using three mouse models. Employing a novel genetic model, combining pancreatic klotho knockdown with a mutation in Kras, the lack of klotho contributed to PDAC generation and decreased mousece survival. In a xenograft model, administration of viral particles carrying sKL, a spliced klotho isoform containing the KL1 domain, inhibited pancreatic tumors. Lastly, treatment with soluble sKL prolonged survival of Pdx1-Cre; Kras^G12D/+^;Trp53^R172H/+^ (KPC) mice, a model known to recapitulate human PDAC. In conclusion, this study provides evidence that klotho is a tumor suppressor in PDAC. Furthermore, these data suggest that the levels of klotho expression and DNA methylation could have prognostic value in PDAC patients, and that administration of exogenous sKL may serve as a novel therapeutic strategy to treat PDAC.

## 1. Introduction

Pancreatic cancer is among the most aggressive cancers, with a 5-year survival rate of 9%. Incidence and mortality are rising, with 60,430 new cases and 48,220 deaths expected in 2021 in the US alone [1]. Pancreatic ductal adenocarcinoma (PDAC) represents 85–90% of all malignant pancreatic neoplasms. Current models indicate its development from benign pancreatic intraepithelial neoplasia (PanIN) 1–3 to invasive carcinoma, along with acquisition of genetic mutations. Activation of Kras is considered the first step towards malignancy [2], and in mouse models, leads to the development of PanIN by 9 months [3]. Although overt PDAC in these mice is rare, in the KPC model, combining Kras mutation with loss of the tumor suppressor p53 [4], PDAC usually develops by 3–6 months of age [5].

Klotho (here used as klotho) is a type I transmembrane protein involved in the regulation of aging [6]. In mice, klotho deficiency leads to a syndrome resembling accelerated aging, while klotho overexpression extends life span [7,8]. The klotho extracellular region is composed of two homologous domains, KL1 and KL2, which can be cleaved from the membrane and act as circulating hormones [9,10,11]. A second, differentially spliced klotho isoform is secreted (sKL). The sKL is identical to KL1, plus 15 additional amino acids at the C-terminus. Different activities of klotho have been described, including activation of fibroblast growth factor (FGF) 23 signaling [12,13], regulation of transient receptor potential cation channel subfamily V member (TRPV) 5 calcium channel [14,15], and inhibition of the insulin and insulin-like growth factor (IGF)-1 pathways [8,16,17].

Klotho is expressed predominantly in the kidneys and brain, but also in various tissues, including the exocrine and endocrine pancreas [7,17,18,19,20]. Its physiologic activities in the pancreas have yet to be determined; however, there is evidence for its involvement in regulation of glucose homeostasis. Klotho induces insulin production and secretion in vitro and in vivo, attenuates insulin sensitivity in mice, and is depleted in pancreatic islets of type 2 diabetes mellitus (T2DM) patients [8,19,20,21,22].

Klotho is a potent tumor suppressor in numerous malignancies, including gastrointestinal cancers [16,17,23,24,25,26,27,28,29,30]. It is epigenetically silenced in cancer, and both klotho and KL1 reduce growth of cancer cells in vitro and in vivo [16,17,23,25,26,28,31,32,33]. In pancreatic cancer, klotho’s downregulation is correlated with patients’ survival. In vitro, klotho reduces growth of pancreatic cancer cells, and inhibits the IGF-I and bFGF pathways [17,34].

In this study, we aimed to decipher the role of klotho as a tumor suppressor in PDAC by utilizing three in vivo models. Employing a novel genetic model, we showed that pancreatic klotho knockdown contributed to PDAC development, and reduced survival of Kras mutant mice. In a second model, we showed that administration of viral particles carrying sKL inhibited pancreatic tumors in a xenograft model. Lastly, treatment with soluble recombinant sKL prolonged survival of KPC mice. These results manifest the role of klotho as an important player in PDAC development, and suggest klotho as a therapeutic strategy to treat PDAC patients.

## 2. Materials and Methods

### 2.1. TCGA Analysis Using UCSC Xena Browser

Gene expression RNAseq (IlluminaHiSeq) and DNA methylation (Methylation450k) datasets for The Cancer Genome Atlas (TCGA) Pancreatic Cancer (PAAD) cohort were studied using the University of California Santa Cruz (UCSC) Xena Browser (http://xena.ucsc.edu/, accessed on 19 July 2020) [35]. Using the Xena Browser, overall survival and the progression-free interval of pancreatic cancer patients were analyzed according to either klotho expression or *KLOTHO* DNA methylation, and Kaplan-Meier plots were created. Duplicated individuals’ data were removed by filtering for primary tumors only. Gene expression data, given in log2 (x + 1) units, were divided into two groups according to median; DNA methylation data were divided into two groups according to lower and upper quartiles.

For site-specific *KLOTHO* DNA methylation, samples with low or high levels of klotho expression, as defined as by lower or upper quartiles, respectively, were further analyzed. The β values [36] for each methylation site were calculated, and correlation with klotho expression in the corresponding sample was examined via Pearson’s correlation coefficient (*r*).

### 2.2. Chemicals, Antibodies, and Constructs

Chemicals used in this study included soluble human splice variant klotho, comprising amino acids 34–549 (sKL domain; accession number BAA24941.1), with a 15-amino-acid unique sequence at the C-terminus (100-53; PeproTech Inc, Rocky Hill, NJ, USA); glucose (Floris, Misgav, Israel), human insulin (Actrapid^®^; Novo Nordisk A/S, Bagsværd, Denmark), and luciferin (122799; PerkinElmer, Waltham, MA, USA).

Antibodies used in this study included rat IgG2a isotype control (02-9688; Thermo Fisher Scientific, Waltham, MA, USA) and anti-β-actin (A5441; Sigma-Aldrich, St. Louis, MO, USA). Anti-klotho antibodies directed against the KL1 domain (KM2076) were a kind gift from Kyowa Hakko Kogyo Co., Ltd., Tokyo, Japan.

### 2.3. AAV Vector Production and Purification

AAV9 vectors, null empty vector, and sKL (containing the secreted klotho splice variant isoform, accession number BAA24941.1) were produced and purified in the biosafety level 2 facilities of the Unitat Mixta UAB-VHIR and the Vector Production Unit (VPU). Briefly, vectors were generated using the triple transfection system in HEK293 cells. After 48 h, AAV vectors were harvested, treated with benzonase, purified in an iodixanol gradient, and titrated using PicoGreen [37]. Transgene expression was driven by a CMV promoter, as previously described [37].

### 2.4. Animal Maintenance

Mouse maintenance and experiments were carried out in the Sourasky Medical Center (Tel Aviv, Israel), and in accordance with the regulations and standards of the Sourasky Medical Center Animal Care and Use Committee.

Mouse strains used for transgenic models were all on a mixed C57BL/6 and JVB/NJ background. Pdx1-Cre, LSL-Kras^G12D/+^, and LSL-Trp53^R172H/+^ mice were kind gifts from Dr. Ziv Gil (Technion-Israel Institute of Technology at Rambam, Haifa, Israel).

### 2.5. Pancreas-Specific KLOTHO Knockdown Mouse Models

In order to target knockdown of *KLOTHO* to mouse pancreata, mice carrying floxed *KLOTHO* alleles (KL^flox^) were generated, as previously described [38]. KL^flox/flox^ mice were crossbred with Pdx1-Cre mice, expressing Cre recombinase controlled by the promotor of Pdx1, thus obtaining Pdx1-Cre; KL^−/−^ mice. Pancreatic *KLOTHO* knockdown was confirmed with immunohistochemistry and mRNA levels, as described hereinafter.

To generate mice harboring Pdx1 promotor-controlled *KLOTHO* knockdown and mutant Kras expression, LSL-Kras^G12D/+^ mice [3], carrying a Lox-Stop-Lox sequence followed by a mutant *Kras* allele, were crossbred with KL^flox/flox^ mice. These mice were further crossbred with Pdx1-Cre mice, yielding Pdx1-Cre; KL^−/−^;Kras^G12D/+^ mice. LSL-Kras^G12D/+^ were also crossbred with Pdx1-Cre mice separately, and these Pdx1-Cre; Kras^G12D/+^ mice were used as the control.

Mice were examined daily for signs of suffering, including hard breathing, major weight changes, and large tumors (more than 1 cm), and those that met the criteria set by the ethical committee were sacrificed. Mice were monitored for survival, with death defined either spontaneously or by signs necessitating sacrifice. Due to the rapid digestion of pancreatic tissue by pancreatic fluids, we could only examine pancreata of mice that had been sacrificed. Tissues were harvested and kept in formaldehyde for 24 h, which was then replaced by 70% ethanol.

### 2.6. Mice Tumor Xenograft Studies

Female athymic nude mice (BALB/c background), 4–6 weeks of age, were purchased from Envigo RMS (Jerusalem, Israel). Mice were housed and maintained in laminar flow cabinets under specific pathogen-free conditions. On the first day of the experiment, MIA PaCa-2 cells stably expressing m-Cherry/luciferase were subcutaneously (s.c.) inoculated into their flanks (*n* = 21, 1 × 10^6^ cells in 100 μL of 5% FCS DMEM medium). Ten days following tumor inoculation, a high dose (5 × 10^11^ GC/mL, *n* = 7) or a low dose (5 × 10^10^ GC/mL, *n* = 6) of human AAV-sKL was injected intramuscularly (i.m.). AAV-null served as the control (5 × 10^10^ GC/mL, *n* = 8). Tumors were measured with a digital caliper three times a week, and volume was calculated by the ellipsoid volume calculation formula (0.5 × length × width^2^). On day 20 of the experiment, tumors were evaluated in vivo by monitoring MIA PaCa-2 cells’ luciferase activity by injection of 150 µg/mL luciferin intra-peritoneally, and luciferase intensity was measured by Biospase.

On the same day, the experiment was ended and the mice were euthanized. Tumors were removed, weighed, and measured with a digital caliper. Blood was collected prior to euthanization, and levels of human klotho were measured in serum using ELISA (IBL, Minneapolis, MN, USA). Fifty microliters of serum per sample (in duplicates) were used for ELISA, and the assay was conducted according to the manufacturer’s instructions. Correlation between weight of tumors and human klotho blood levels was examined via Pearson’s correlation coefficient.

### 2.7. KPC Mouse Models

Generation of KPC mice was done by crossbreeding LSL-Trp53^R172H/+^ mice [5] carrying a Lox-Stop-Lox sequence, followed by a point-mutant p53 allele that functions as a null mutation, with LSL-Kras^G12D/+^ mice. These were further crossbred with Pdx1-Cre mice, resulting in Pdx1-Cre; Kras^G12D/+^; Trp53^R172H/+^ mice. Mice were matched according to sex, age, and weight, and were randomly assigned to receive treatment with intraperitoneal (i.p.) injections of soluble human sKL (15 mg/kg, twice weekly) or a vehicle control (*n* = 6 for the sKL-treated group; *n* = 7 for the control group). Treatment continued for up to 30 weeks. Mice were examined daily for signs of suffering, including hard breathing, major weight changes, and large tumors (more than 1 cm), and those that met the criteria set by the ethical committee were sacrificed. Mice were monitored for survival, with death defined either spontaneously or by signs necessitating sacrifice.

### 2.8. Genotyping

Mouse tails were taken at 3 weeks of age for genotyping. DNA was extracted by incubation of the tail in alkaline lysis solution, consisting of 250 µM NaOH and 2 µM disodium EDTA (pH 12.0), for 30–60 min in 95 °C, followed by neutralization with 0.4 mM Tris HCL (pH 5.0). DNA subsequently underwent PCR amplification for the different genotypes, using REDTaq ReadyMix PCR Reaction Mix (Sigma-Aldrich, Rehovot, Israel). Primers were at 1000 nM final concentration. PCR cycles and primers are detailed in Table S1. Products were electrophoresed on 1.5% agarose gels and stained with ethidium bromide.

### 2.9. Immunohistochemistry (IHC) Analysis

Tissues were fixed in 4% paraformaldehyde, and embedded tissues were serially sectioned. Sections were either stained with hematoxylin and eosin (H&E) and examined microscopically by an experienced pathologist, or stained by IHC, as described.

For klotho IHC, formalin-fixed and paraffin-embedded sections, 4 μm thick, were dewaxed in xylene and rehydrated. Antigen retrieval was performed using a hot bath (95 °C) for 20 min in citrate buffer pH 6.0. After cooling for 30 min, slides were rinsed in TBS-triton (TBS-T) buffer. Subsequently, an endogenous peroxidase block was performed for 10 min in 3% H_2_O_2_/methanol. After rinsing in TBS-T, sections were blocked for 30 min using 5% BSA in TBS-T, and later were incubated overnight with klotho primary antibody at 4 °C. Detection was performed with ZytoChem Plus (HRP) One-Step Polymer anti-Mouse/Rabbit/Rat (ZUC053; Zytomed Systems, Berlin, Germany). Rat IgG2a served as the isotype control.

### 2.10. Reverse Transcription Polymerase Chain Reaction (RT-PCR)

Pancreata from sacrificed Pdx1-Cre; KL^−/−^ and KL^flox/flox^ control mice were excised, frozen in liquid nitrogen, and stored at −80 °C. Following homogenization of the pancreata, total RNA was prepared using the RNA isolation kit (Sigma), and reverse transcribed using qScript cDNA SuperMix (Quanta BioSciences, Gaithersburg, MD, USA). The cDNA was amplified for klotho and β-actin (as the loading control) using REDTaq ReadyMix PCR Reaction Mix (Sigma). The PCR was optimized at 94 °C for 5 min, followed by cycles of 30 s at 94 °C, 90 s at 52.5 °C, and 20 s at 72 °C (45 cycles for klotho, 25 cycles for β-actin), and 10 min at 72 °C for extension, using 12.5 pmol klotho primers (F, 5′-ACGTTCAAGTGGACACTACTCT-3′ and R, 5′- TTCTTGGCTACAACCCCGTC-3′) and 5 pmol β-actin primers (F, 5′-TGTTACCAACTGGGACGACA-3′ and R, 5′- GGGGTGTTGAAGGTCTCAAA-3′). Products were electrophoresed on 1.5% agarose gels stained with ethidium bromide. Quantification was done using ImageJ software, National Institutes of Health, Bethesda, MD, USA.

### 2.11. Statistical Analysis

Results are presented as the mean ± SD or SEM, as mentioned. Continuous variables were compared using the *t* test, unless otherwise mentioned. All significance tests were two-tailed, and a *p* value of ≤0.05 was considered as statistically significant. Correlation was assessed using Pearson’s correlation coefficient (*r*). Survival analysis was done using the log-rank test.

## 3. Results

### 3.1. Levels of Klotho Expression and DNA Methylation in Pancreatic Tumors Correlate with Survival of Cancer Patients

**Figure 1:**
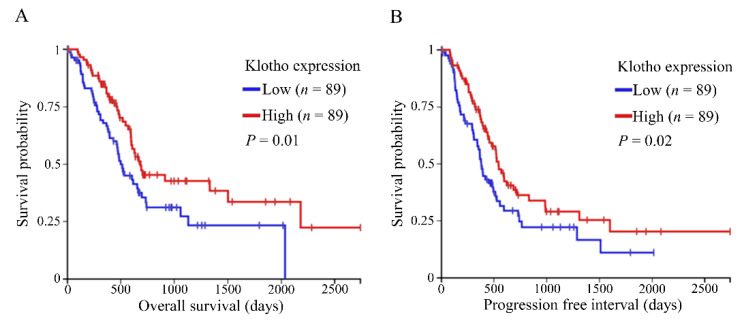
Low klotho expression reduces survival of pancreatic cancer patients. (**A**,**B**) The UCSC Xena Browser was used to examine The Cancer Genome Atlas (TCGA) Pancreatic Cancer (PAAD) cohort. Gene expression RNAseq (IlluminaHiSeq) dataset was analyzed for overall survival, and progression-free interval of patients with low (<5.675, *n* = 89) vs. high (≥5.675, *n* = 89) klotho expressing tumors.

**Figure 2:**
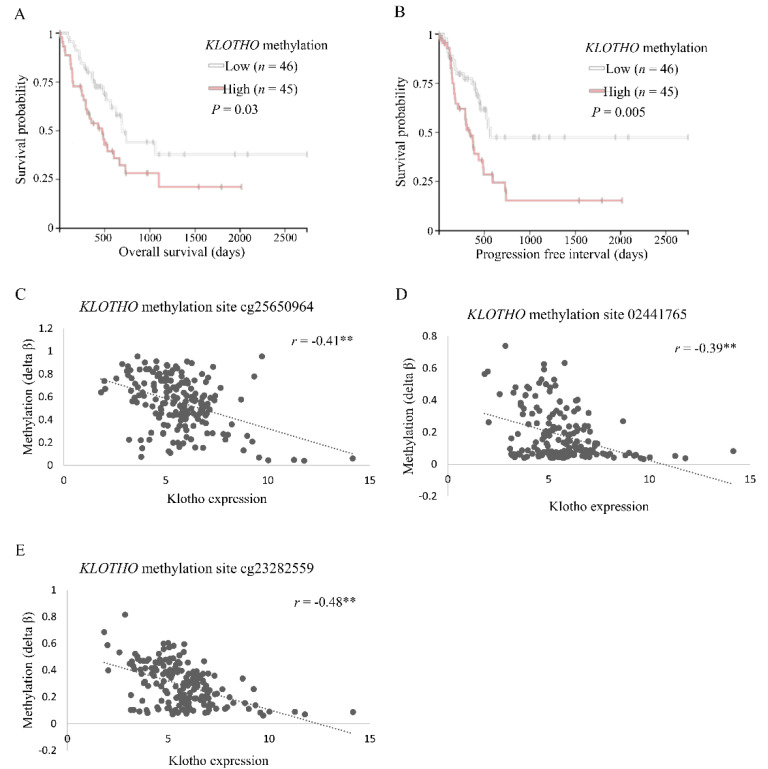
*KLOTHO* is hypermethylated in pancreatic cancer. (**A**–**E**) The UCSC Xena Browser was used to examine TCGA PAAD cohort. (**A**,**B**) RNA Methylation450k dataset was analyzed for overall survival and progression-free interval of patients with tumors showing low (<0.5152, *n* = 46) vs. high (>0.5830, *n* = 45) *KLOTHO* methylation. (**C**–**E**) Gene expression RNAseq (IlluminaHiSeq) and RNA Methylation450k datasets were analyzed for correlation between klotho expression and methylation at the indicated methylation sites (*n* = 178). ** *p* < 0.005. *r*, Pearson’s correlation coefficient.

The Cancer Genome Browser (TCGA) Pancreatic Cancer (PAAD) cohort was examined using the University of California Santa Cruz (UCSC) Xena Browser [35]. Gene expression analyses revealed reduced overall survival (OS) and a progression-free interval (PFI) for pancreatic cancer patients with low klotho expressing tumors, compared to high (*n* = 89 for each group; Figure 1A,B). Examination of methylation data showed a compatible trend of hampered OS and PFI in patients who had tumors with high *KLOTHO* DNA methylation levels, compared to low (*n* = 45 and *n* = 46, respectively; Figure 2A,B).

Further analyses of gene expression and methylation data (Table S2) revealed three specific sites, evaluated by probes cg23282559, cg02441765, and cg25650964, which were hypermethylated (|Delta *β*| > 0.15) and negatively correlated to klotho expression (|*r*| > 0.3) in human pancreatic cancer (Figure 2C–E).

### 3.2. Generation of Pancreatic KLOTHO Knockdown Mice

**Figure 3:**
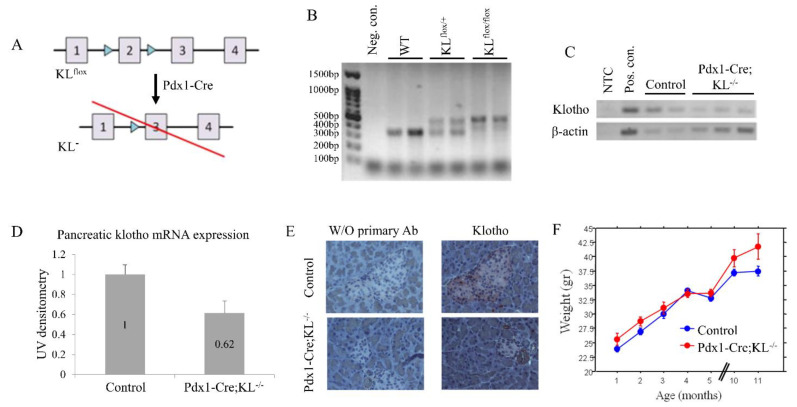
Generation of pancreatic *KLOTHO* knockdown mice. (**A**) Schematic diagram of KL^flox^ allele: LoxP sites flank exon 2 of *KLOTHO*, leading to its targeted knockdown in pancreata of Pdx1-Cre; KL^−/−^ mice. Top panel shows floxed allele, bottom panel shows expected results of Cre recombination. (**B**) Representative gel showing PCR products of mouse *KLOTHO* genotyping: KL^+/+^ (WT; 370 bp), KL^flox/+^ (470 and 370 bp), and KL^flox/flox^ (470 bp). (**C**,**D**) Pancreatic RNA was extracted from Pdx1-Cre; KL^−/−^ (*n* = 7) and control mice (*n* = 4). Klotho mRNA levels were determined by semi-quantitative RT-PCR and quantified. Representative gel shown (original uncropped blots are available as Figure S2). (**E**) Representative immunohistochemical klotho staining of pancreata excised from Pdx1-Cre; KL^−/−^ and control mice. Magnification: X20. W/O, without. (**F**) Weight of mice in both groups (*n* = 5 per group). Analyzed using repeated-measures ANOVA. *p* = 0.01. Data are presented as the mean ± SEM. Control, KL^flox/flox^ mice.

Non-conditional *KLOTHO* knockout mice die at around 8–9 weeks of age [7], hence, they cannot serve as models for cancer development. Therefore, we aimed to target *KLOTHO* knockdown to mice pancreata. For this aim, Pdx1-Cre mice were crossbred with mice carrying floxed *KLOTHO* alleles, KL^flox/flox^ [38], thus obtaining Pdx1-Cre; KL^−/−^ mice (Figure 3A,B). RNA and protein levels validated pancreatic klotho knockdown (Figure 3C–E). Pdx1-Cre; KL^−/−^ mice showed a slight increase in weight over time, compared to the control (Figure 3F), but no differences in serum glucose or insulin and glucose tolerance (Figure S1).

Mice harboring pancreatic *KLOTHO* knockdown and mutant Kras expression were generated by crossbreeding Pdx1-Cre with LSL-Kras^G12D/+^ [3] mice (Pdx1-Cre; Kras^G12D/+^ mice), and further crossbreeding them with KL^flox/flox^ mice (Pdx1-Cre; KL^−/−^; Kras^G12D/+^ mice). Analyses were conducted using male mice. Female mice were excluded, due to a high rate of anal lesions in both Pdx1-Cre; Kras^G12D/+^ and Pdx1-Cre; KL^−/−^; Kras^G12D/+^ mice, which is consistent with previous reports of mucocutaneous papillomas in such mice [3].

### 3.3. Loss of Pancreatic Klotho Contributes to Reduced Survival in Mice

**Figure 4:**
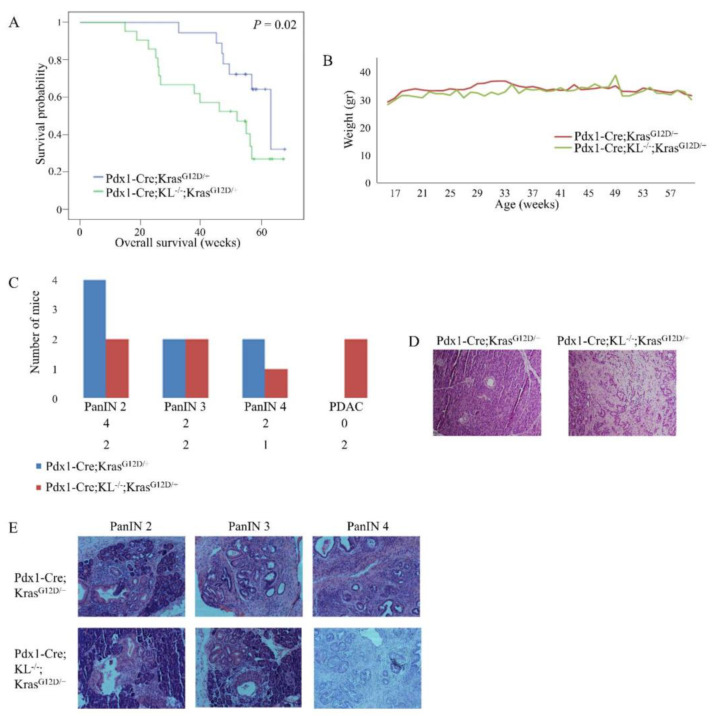
Pancreatic *KLOTHO* knockdown contributes to reduced survival and induces PDAC in vivo. (**A**) Kaplan-Meier curves of Pdx1-Cre; KL^−/−^; Kras^G12D/+^ (*n* = 21) compared to control Pdx1-Cre; Kras^G12D/+^ (*n* = 18) mice. *p* = 0.02. (**B**) Weight of mice in both groups (*n* = 19 per group). (**C**–**E**) Pancreata were excised from Pdx1-Cre; KL^−/−^; Kras^G12D/+^ (*n* = 7, average age 36 weeks) and control Pdx1-Cre; Kras^G12D/+^ (*n* = 8, average age 49 weeks) mice upon sacrifice. (**C**) Comparison between pathological assessment of H&E stained pancreata of both groups. (**D**) Representative H&E staining of pancreata harvested from 24-week-old mice. Pdx1-Cre; KL^−/−^; Kras^G12D/+^ (right): complete loss of cellular polarity, significant nuclear atypia, and budding of cell clusters into the ductal lumen, along with profuse fibrosis and keratin. Control Pdx1-Cre; Kras^G12D/+^ (left): no lesions. Magnification: ×10. (**E**) Representative PanIN lesions of both groups. Magnification: ×10.

There was no difference in survival of Pdx1-Cre; KL^−/−^ mice, compared to the control). We proceeded to study the effect of combined pancreatic *KLOTHO* knockdown and Kras mutation on survival by monitoring the mice for 65 weeks. Death occurred either spontaneously or by euthanasia when signs of severe suffering, or tumor burden that necessitated sacrifice, started. Survival of Pdx1-Cre; KL^−/−^; Kras^G12D/+^ mice (*n* = 21) was decreased, compared to the control Pdx1-Cre; Kras^G12D/+^ mice (*n* = 18; *p* = 0.02; Figure 4A). Thus, by the age of 22 weeks, 100% (18/18) of Pdx1-Cre; Kras^G12D/+^ mice were alive, compared to 71% (15/21) of Pdx1-Cre; KL^−/−^; Kras^G12D/+^ mice. No significant changes in weight were noted between the groups (Figure 4B).

### 3.4. Klotho Cooperates in the Development of PDAC In Vivo

Based on previous studies showing PDAC development in mice harboring both Kras mutation and loss of tumor suppressor activity [4], we examined Pdx1-Cre; KL^−/−^; Kras^G12D/+^ mouse pancreata for lesions. Due to the rapid digestion of pancreatic tissue by pancreatic fluids, we could only use pancreata of mice that had been sacrificed. Samples of Pdx1-Cre; KL^−/−^; Kras^G12D/+^ (*n* = 7) and control Pdx1-Cre; Kras^G12D/+^ (*n* = 8) mice underwent pathological evaluation (average age of death: 36 and 49 weeks, respectively). PDAC was identified in two Pdx1-Cre; KL^−/−^; Kras^G12D/+^ mice, but in none of the Pdx1-Cre; Kras^G12D/+^ mice, while PanIN 2 was noted in two of the Pdx1-Cre; KL^−/−^;Kras^G12D/+^ mice and in four of the Pdx1-Cre;Kras^G12D/+^ mice (Figure 4C). Representative images are shown (Figure 4D–E).

### 3.5. Treatment with sKL Inhibits Pancreatic Tumors and Prolongs Survival In Vivo

**Figure 5:**
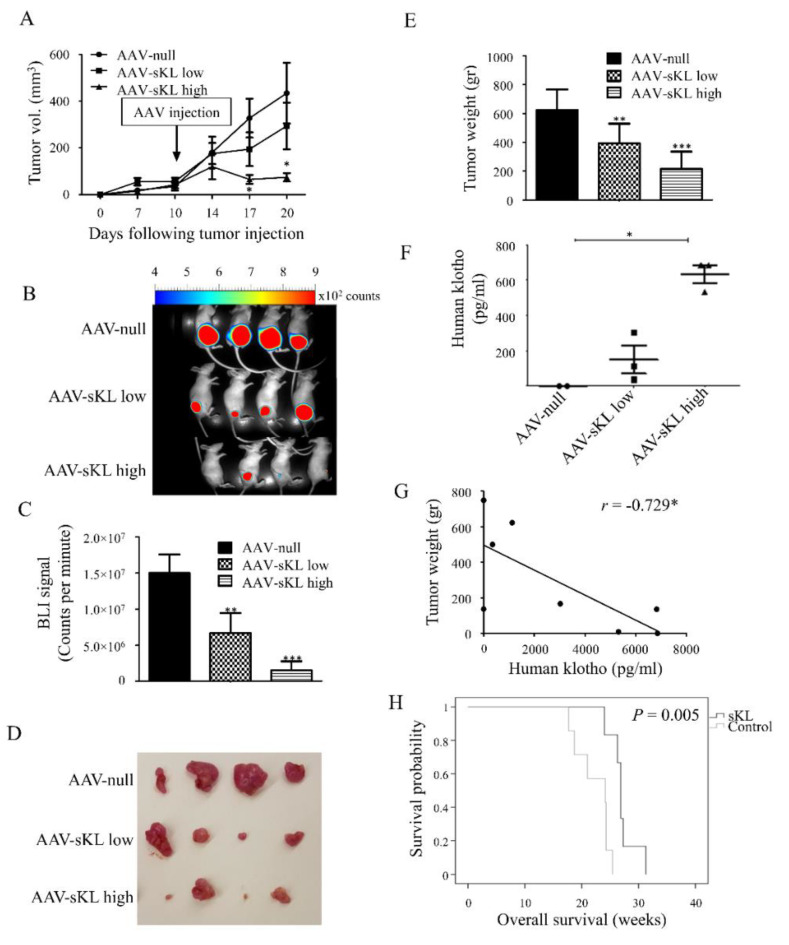
sKL treatment decreases local tumor growth and prolongs survival in vivo. (**A**–**G**) Athymic nude BALB/c mice were s.c. inoculated with MIA PaCa-2 cells stably expressing m-Cherry/luciferase (1 × 10^6^ cells per mouse). Ten days later, mice were injected i.m. with high dose AAV-sKL (5×10^11^ GC/mL, *n* = 7), low dose AAV-sKL (5 × 10^10^ GC/mL, *n* = 6), or control AAV-null (5 × 10^10^ GC/mL, *n* = 8). (**A**) Tumor volume was measured in vivo with a digital caliper. (**B**) Representative pictures of luciferase activity bioimaging of the local tumors. (**C**) Tumors’ luciferase activity was measured by counts per minute. (**D**) Representative images of tumors harvested on day of sacrifice. (**E**) Weight of harvested tumors. (**F**) Human klotho blood levels. * *p* < 0.05, was calculated using Kruskal-Wallis test. (**G**) Correlation between tumor weights and human klotho blood levels. * *p* < 0.05. *r*, Pearson’s correlation coefficient. (**H**) KPC mice were matched according to sex, age, and weight, and randomly assigned to receive treatment with i.*p*. injections of soluble human sKL (15 mg/kg, twice weekly) or a vehicle control (*n* = 6 for the sKL-treated group; *n* = 7 for the control group) for up to 30 weeks. Kaplan-Meier curves of sKL-treated and control mice are presented. *p* = 0.005. * *p* < 0.05; ** *p* < 0.005; *** *p* < 0.0005 compared to control. Error bars, mean ± SEM.

The potential of sKL treatment as a therapeutic strategy for PDAC was studied using two mouse models, a xenograft model treated with a viral sKL vector, and the transgenic KPC model treated with soluble, recombinant sKL. For the xenograft model, m-Cherry/luciferase-labeled MIA PaCa-2 cells were s.c. inoculated into nude mice. Ten days later, mice were injected with adeno-associated viruses (AAV) encoding sKL (AAV-sKL) at two doses. Tumor load in mice inoculated with AAV-sKL was significantly lower compared to the control, reflected by smaller size, weight, and luciferase signals (Figure 5A–E). Importantly, tumor weights negatively correlated with human klotho blood levels in mice (|*r*| > 0.729; Figure 5F–G).

The second model utilized for this aim was the KPC model. Mice were matched according to sex, age, and weight, and treated with i.p. injections of soluble human sKL or a vehicle control for up to 30 weeks. Death occurred either spontaneously or by euthanasia when signs of severe suffering, or tumor burden that necessitated sacrifice, started. Survival of mice treated with sKL was increased, compared to the control (*p* = 0.005; Figure 5H).

## 4. Discussion

The present study establishes the role of klotho as a tumor suppressor in PDAC. Bioinformatics analyses revealed a correlation between the survival of PDAC patients, and levels of klotho expression and DNA methylation, and demonstrated a unique hypermethylation pattern of *KLOTHO* in pancreatic tumors. Using a novel mouse model, we showed that pancreatic klotho knockdown cooperates with Kras mutation to decrease survival and generate PDAC in vivo. Moreover, sKL inhibited growth of tumors originating from pancreatic cells in a xenograft model, and prolonged survival of KPC mice.

Klotho is epigenetically silenced through promoter hypermethylation in a wide array of malignancies [16,23,25,26,28,29,30,31,33,39,40]. This has also been reported in small cohorts in PDAC, as well as in a correlation between survival of PDAC patients and levels of klotho expression and DNA methylation [17,34]. In accordance, our evaluation of OS, as well as PFI using data comprising 178 PDAC samples, showed a positive association with klotho expression and a corresponding negative association with *KLOTHO* DNA methylation. These data further indicate that klotho expression may be regulated by DNA methylation, and reinforce the notion that these parameters could serve to assess the prognosis of PDAC patients.

Previous studies of *KLOTHO* DNA methylation in PDAC [17,34] were based on a limited subset of patients and in vitro experiments, and did not analyze the methylation pattern of specific sites within the entire *KLOTHO* gene. The current analysis, based on the aforementioned extensive dataset, revealed that three specific sites, two of which are located within a CpG island in the 1st exon of *KLOTHO*, act as negative regulators of klotho expression by hypermethylation. We previously found that one of these sites, cg23282559, is also hypermethylated in colorectal cancer [25]. It is yet to be determined whether these sites play a role in klotho regulation in other malignancies as well.

Klotho is known to be expressed in the pancreas; however, its role in normal pancreatic development and function is still unknown. While klotho-deficient mice show decreased insulin production, along with a dramatic increase in insulin sensitivity [22], overexpression of klotho in mice results in increased fasting blood insulin, accompanied by insulin resistance [8]. Surprisingly, the mouse model presented in this study, harboring pancreatic klotho knockdown, did not show an overt phenotype of diabetes or altered sensitivity to insulin. Furthermore, there was no effect on pancreatic morphology, tumor formation, nor survival. These results suggest that loss of pancreatic klotho may be compensated by peripheral effects of systemic klotho or by other mechanisms. Moreover, diabetes development is a complex process, which depends not only on the pancreas, but also on the liver, muscles, and fat tissues. As in our model, klotho was knockdown mainly in the pancreas, it may explain the lack of diabetes in this mouse model.

Consistent with the clinical results presented, Pdx1-Cre; KL^−/−^; Kras^G12D/+^ mice had shortened life spans compared to the control Pdx1-Cre; Kras^G12D/+^ mice (48 vs. 60 weeks, respectively). Although we were unable to pathologically examine all the mouse pancreata, a higher rate of overt PDAC was seen in Pdx1-Cre; KL^−/−^; Kras^G12D/+^ mice, compared to the control Pdx1-Cre; Kras^G12D/+^ mice. These results demonstrate the significance of klotho in the development of pancreatic malignancies and survival in vivo. The phenotype of Pdx1-Cre; KL^−/−^;Kras^G12D/+^ mice is less pronounced, compared to models combining loss of other tumor suppressors with Kras mutations. As an example, KPC mice have a median survival of only 5 months [5]. It is possible that this is due to partial compensation of loss of local klotho by circulating klotho. Alternatively, residual expression of klotho in the pancreas may have affected the phenotype. Achieving complete targeted silencing of klotho in future studies would enable a better understating of its role in different tissues, as well as the counterbalance between local and systemic effects of klotho.

We presented two models demonstrating the therapeutic potential of klotho. In a xenograft mouse model, pancreatic cancer injections of a viral sKL vector were highly effective, not only in inhibiting tumor growth, but also in reducing the size of tumors. Treatment began when tumors were already visible and established, resembling the time of treatment initiation in most patients. The results suggest that sKL may be utilized for gene therapy, thus bypassing major obstacles of stability and efficacious delivery of protein and peptide-based drugs. Moreover, a negative correlation between tumor weight and human klotho blood levels was noted. Further research of klotho blood levels in respect to its effect may assist in predicting who would benefit from treatment with klotho or sKL, as well as in keeping levels within the therapeutic window.

The development of klotho-based therapies is a goal sought by many research groups, as well as pharmaceutical companies. In regard to cancer treatment, there are several avenues that are explored. One can increase the endogenous levels of klotho by taking advantage of epigenetic mechanisms shown to affect klotho expression. It has been reported that the klotho promoter is heavily methylated in many cancers, and is also a function of age. Thus, compounds that inhibit DNA methyl transferases could become a therapeutic target to increase klotho expression. On the other hand, one can increase the levels by adding klotho exogenously, for example, by employing gene therapy or delivery of a recombinant klotho protein. Here, we reported the introduction of the active region of klotho (sKL) using the AAV expression system, which resulted in a therapeutic effect in a model of pancreatic cancer.

Next, we showed that treatment with soluble sKL prolonged survival of KPC mice, a model known to recapitulate human PDAC. This demonstrated the effect of sKL in an immune-competent host and native tumor environment, further supporting the possible use of sKL in clinical settings.

## 5. Conclusions

In conclusion, this study identified klotho as a potent tumor suppressor in PDAC. Current treatment regimens for PDAC are not sufficient, and administration of exogenous sKL may serve as a novel strategy for the treatment of PDAC.

## Data Availability

The data presented in this study are available on request from the corresponding author.

## Supplementary Materials

The following are available online at https://www.mdpi.com/article/10.3390/cancers13246297/s1, Figure S1: Pancreatic klotho knockdown does not affect insulin sensitivity in mice, Figure S2: Original uncropped blots of klotho (**A**) and β-actin (**B**) from Figure 3C, Table S1: PCR cycles and primers’ sequences used for genotyping, Table S2: Differential methylation, correlation with klotho expression, and characteristics of *KLOTHO* DNA methylation sites.
