# Supplementary material for: A Transgenic Model Reveals the Role of Klotho in Pancreatic Cancer Development and Paves the Way for New Klotho-Based Therapy
Source: Cancers (Basel). 2021 Dec 15;13(24):6297. doi: 10.3390/cancers13246297 (PMC8699737; doi:10.3390/cancers13246297)
Supplement: Supplementary file 1 [file cancers-13-06297-s001.zip › cancers-1485297-supplementary.pdf]

Supplementary Materials: A Transgenic Model Reveals the Role of Klotho in Pancreatic Cancer Development and Paves the Way for New Klotho-Based Therapy

Tammi Arbel Rubinstein, Inbal Reuveni, Arkadi Hesin, Anat Klein-Goldberg, Hannes Olauson, Tobias E. Larsson, Carmela R. Abraham, Ella Zeldich, Assumpció Bosch, Miguel Chillón, Kenneth Samuel Hollander, Ayelet Shabtay-Orbach, Gilad W. Vainer, Ido Wolf and Tami Rubinek

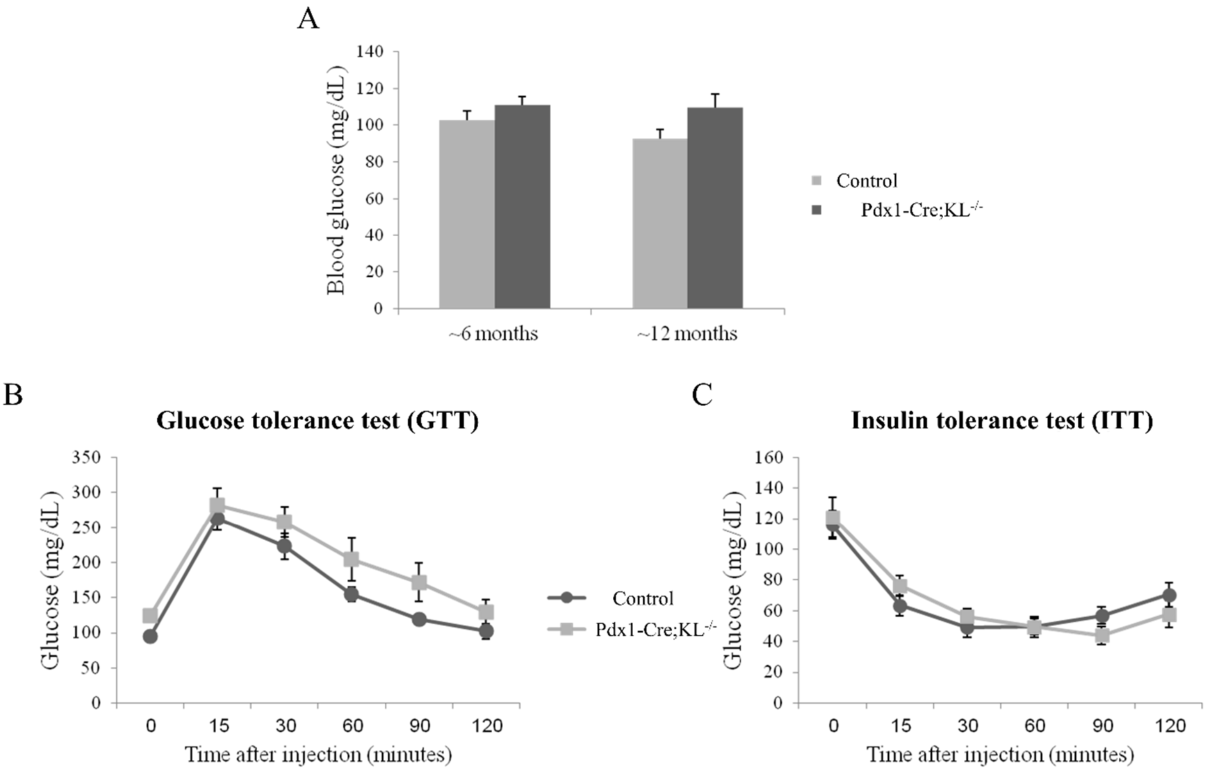

Figure S1. Pancreatic klotho knockdown does not affect insulin sensitivity in mice. (**A**) Blood glucose levels of Pdx1-Cre;KL$^{-/-}$ and control KL$^{flox/flox}$ mice, ages 6- and 12-months. **(B,C)** GTT and ITT of Pdx1-Cre;KL$^{-/-}$ and control KL$^{flox/flox}$ mice. Results are the mean ± SEM.

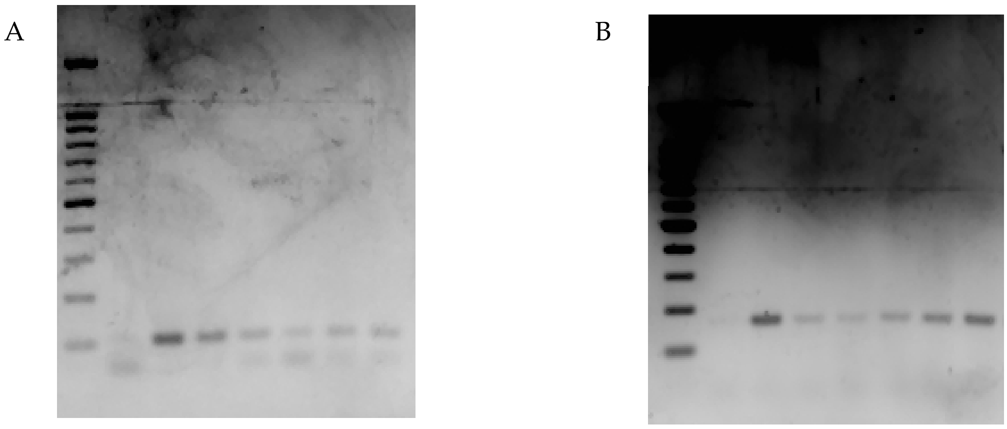

Figure S2. Original uncropped blots of klotho (**A**) and β-actin (**B**) from figure 3C.

Table S1. PCR cycles and primers' sequences used for genotyping.

Target Gene	Primers' Sequences	WT Amplicon (bp)	Mutant Amplicon (bp)	PCR cycle
Klotho	F: 5'-TTGTCAATATGTAAATAATTTGAGCAGTAGGG-3'<br>R: 5'-GTTGTTGAAAGAGGGAGCTAGTGGTAGTTA-3'	370	470	94°C–3 min<br>94°C–30 s *<br>58°C–30 s *<br>72°C–1 min *<br>72°C–3 min
Kras	Mutant F: 5'-CTAGCCACCATGGCTTGAGT-3'<br>WT F: 5'-ATGTCTTTCCCCAGCACAGT-3'<br>R: 5'-TCCGAATTCAGTGACTACAGATG-3'	450	327	94°C–3 min<br>94°C–30 s *<br>58.5°C–30 s *<br>72°C–1 min *<br>72°C–7 min
p53	Mutant F: 5'-AGCTAGCCACCATG GCTTGAGTAAGTCTGCA-3'<br>WT F: 5'-TTACACATCCAGCCTCTGTGG-3'<br>R: 5'-CTTGGAGACATAGCCACACTG-3'	166	270	
Cre	F: 5'-CTGGACTACATCTTGAGTTGC-3'<br>R: 5'-GGTGTACGGTCAGTAAATTTG-3'	-	650	

bp, base pairs; F, forward; R, reverse; WT, wild-type; *, 35 cycles.

Table S2. Differential methylation, correlation with klotho expression and characteristics of *KLOTHO* DNA methylation sites.

Probe	DNA Location (hg19)	UCSC Ref-Gene Group	Location within CpG Island	Delta β	Correlation (*r*)
cg10288525	chr13:33455186-33455188			−0.04	0.15*
cg19133973	chr13:33467979-33467981			−0.01	0.03
cg11668133	chr13:33477098-33477100			0.01	−0.02
cg22811384	chr13:33528604-33528606			−0.02	0.10
cg25650964	chr13:33544013-33544015			0.21	−0.41**
cg05132118	chr13:33580951-33580953			−0.01	0.14
cg18056695	chr13:33589284-33589286	TSS1500	+	0.08	−0.09
cg23943268	chr13:33589620-33589622	TSS1500	+	0.06	−0.23**
cg17806623	chr13:33590001-33590003	TSS1500	+	0.08	−0.28**
cg09886946	chr13:33590047-33590049	TSS1500	+	0.12	−0.40**
cg21545902	chr13:33590082-33590084	TSS1500	+	0.12	−0.37**
cg05855588	chr13:33590272-33590274	TSS1500	+	0.10	−0.23**
cg17106222	chr13:33590342-33590344	TSS1500	+	0.05	−0.23**
cg25698998	chr13:33590400-33590402	TSS200	+	0.02	−0.19*
cg02796545	chr13:33590465-33590467	TSS200	+	0.12	−0.32**
cg23132624	chr13:33590472-33590474	TSS200	+	0.11	−0.25**
cg14145477	chr13:33590492-33590494	TSS200	+	0.10	−0.23**
cg05116906	chr13:33590494-33590496	TSS200	+	0.09	−0.21**
cg02441765	chr13:33590837-33590839	1st exon	+	0.16	−0.39**
cg23282559	chr13:33591129-33591131	1st exon	+	0.17	−0.48**
cg12162530	chr13:33591782-33591784	Gene body	+	0.05	−0.27**
cg01308409	chr13:33591936-33591938	Gene body	+	0.11	−0.36**
cg20672059	chr13:33594283-33594285	Gene body	+	−0.01	0.05
cg23584087	chr13:33626628-33626630	Gene body		−0.04	0.20*
cg26325430	chr13:33639058-33639060	3'UTR		−0.03	0.16*
cg13415069	chr13:33640532-33640534			−0.02	0.24**
cg09634936	chr13:33640667-33640669			0.00	0.06
cg11091909	chr13:33640725-33640727			0.00	0.07
cg01463226	chr13:33640867-33640869			0.00	0.04
cg08416394	chr13:33640928-33640930			0.00	0.01
cg19860320	chr13:33640941-33640943			0.01	−0.15*
cg00069969	chr13:33646762-33646764			−0.02	0.15*

*, $p<0.05$; **, $p<0.005$. CpG Island, UCSC CpG Island chr13:33589928-33591428; Correlation (*r*), Pearson's correlation coefficient; Delta β, $\beta_{Tumor}-\beta_{Normal\ Tissue}$; TSS1500, region from -1500 to -200 nucleotides upstream of *KLOTHO* start site; TSS200, region from -200 nucleotides upstream to the transcription site itself.

Materials and methods

Serum glucose measuring

Following a 6 hour fast, glucose levels were measured in tail blood of

Pdx1-Cre;$KL^{-/-}$ and $KL^{flox/flox}$ mice, ages 6-8 and 11-13 months (later referred to as 6- and 12-months-old, respectively), using FreeStyle Lite® glucometer.

Glucose tolerance test (GTT) and insulin tolerance test (ITT)

Following an overnight fast, Pdx1-Cre;$KL^{-/-}$ and $KL^{flox/flox}$ mice were challenged with intraperitoneal (IP) injections of either 2 g/kg glucose (Floris, Misgav, Israel) or 0.5 U/kg human insulin (Actrapid®; Novo Nordisk A/S, Bagsværd, Denmark), respectively. Glucose levels were measured in tail blood at 15, 30, 60, 90 and 120 minutes from injection.

Results

Loss of pancreatic klotho does not alter glucose levels, glucose or insulin tolerance

Pdx1-Cre;$KL^{-/-}$ and control $KL^{flox/flox}$ mice were tested for fasting glucose as well as GTT and ITT. There were no significant differences between the groups (Supplementary Figure S1).
